# Gradual Optimization of Molecular Aggregation and Stacking Enables Over 19% Efficiency in Binary Organic Solar Cells

**DOI:** 10.1002/advs.202409867

**Published:** 2024-10-02

**Authors:** Jianqiang Qin, Linze Wu, Sihao Huang, Zeping Ou, Xiaowu Wang, Yingguo Yang, Yujie Zheng, Kuan Sun, Zeyu Zhang, Zhiping Hu, Zhengzheng Liu, Yuxin Leng, Juan Du

**Affiliations:** ^1^ School of Physics and Optoelectronic Engineering Hangzhou Institute for Advanced Study University of Chinese Academy of Sciences Hangzhou 310024 China; ^2^ State Key Laboratory of High Field Laser Physics and CAS Center for Excellence in Ultra‐intense Laser Science Shanghai Institute of Optics and Fine Mechanics (SIOM) Chinese Academy of Sciences (CAS) Shanghai 201800 China; ^3^ MOE Key Laboratory of Low‐grade Energy Utilization Technologies and Systems, School of Energy & Power Engineering Chongqing University Chongqing 400044 China; ^4^ School of Microelectronics Fudan University Shanghai 200433 China

**Keywords:** morphology control, organic solar cells, solid additive, solvent vapor annealing

## Abstract

Volatile solid additive is an effective and simple strategy for morphology control in organic solar cells (OSCs). The development of environmentally friendly new additives which can also be easily removed without high‐temperature thermal annealing treatment is currently a trend, and the working mechanism needs to be further studied. Herein, a highly volatile and non‐halogenated solid additive 1‐benzothiophene (BBT) is reported to regulate molecular aggregation and stacking of active layer components. According to the film‐forming kinetics process, a momentary intermediate phase is formed during spin‐coating, which slows down the film‐forming process and leads to more ordered molecular stacking in the solid film after introducing solid additive BBT. Subsequently, after solvent vapor annealing (SVA) further treatment, the resultant blend films exhibit a tighter and more ordered molecular stacking. Consequently, the synergistic effect of solid additive BBT and SVA treatment can effectively control morphology of active layer and improve carrier transport characteristics, thereby enhancing the performance of OSCs. Finally, in D18‐Cl:N3 system, an impressive power conversion efficiency of 19.53% is achieved. The work demonstrates that the combination of highly volatile solid additives and SVA treatment is an effective morphology control strategy, guiding the development of efficient OSCs.

## Graphical abstract

An environmental‐friendly (halogen‐free) 1‐benzothiophene (BBT) is employed as solid additive and combined it with solvent vapor annealing (SVA) post‐treatment to optimize the morphology of active layer and improve performance of OSCs. Ultimately, an impressive efficiency of 19.53% is obtained in D18‐Cl:N3 OSCs, which is one of the highest reported efficiencies for binary OSCs to date.

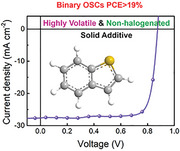

## 1 Introduction

Energy shortages and environmental pollution continue to plague humanity, so the development of renewable energy has become increasingly important. Recently, organic solar cells (OSCs) have attracted immense attention in the field of photovoltaic technology. With the continuous emergence of high‐performance polymer donors and non‐fullerene acceptors, the power conversion efficiency (PCE) of state‐of‐the‐art OSCs has exceeded 19%.^[^ ^1^ ^]^ It is generally recognized that morphology of active layer can effectively affect the exciton generation and dissociation, charge transport, and charge collection processes, which can directly determine performance of devices. Therefore, the ideal morphology of the active layer with proper phase separation, appropriate domain size, high domain purity, and excellent molecular stacking is one of the key characteristics to achieving high performance.^[^ ^2^ ^]^ To date, plenty of strategies have been proposed to control morphology of active layer, such as optimizing molecular structure, solvent vapor annealing (SVA), thermal annealing, ternary and quaternary strategy, and solvent and solid additives, etc.^[^ ^3^ ^]^

Among them, additive strategy is an effective and simple method for morphology control.^[^ ^4^ ^]^ Moreover, compared with the solvent additive with high boiling point, volatile solid additive is relatively insensitive to additive amount and will not remain in the active layer, resulting in high reproducibility and stability.^[^ ^5^ ^]^ Considering this, many efforts have been made to optimize morphology of active layer and improve performance of OSCs by using the volatile solid additive. Hou et al. designed and synthesized a series of volatile solid additives with a similar structure of the end‐groups of non‐fullerene acceptors to enhance the *π–* *π* stacking of the non‐fullerene acceptors and thus improve carrier transport property, resulting in increased PCE of OSCs.^[^ ^6^ ^]^ Subsequently, Lu et al. reported a simple solid additive 1,4‐diiodobenzene (DIB) to control morphology of the active layer with tighter and more ordered molecular stacking, leading to improved performance and stability of OSCs. Moreover, solid additive DIB has excellent versatility in polymer and all‐small‐molecule OSCs.^[^ ^7^ ^]^ Subsequently, a series of solid additives with similar structure to DIB, such as 3,5‐dichlorobromobenzene (DCBB), 1,4‐dibromobenzene (PDBB), and 1,3,5‐trichlorobenzene (TCB), have been successfully reported and applied in OSCs.^[^ ^8^ ^]^ These works demonstrate that volatile solid additives, which have significant advantages in morphology control, remain a hot topic in the field of OSCs. However, most of the reported volatile solid additives contain halogens. Meanwhile, in reports related to volatile solid additives, high‐temperature thermal annealing treatment is necessary to provide driving force for solid additive removal and molecular rearrangement, but it is not conducive to the preparation of flexible devices.^[^ ^9^ ^]^ Moreover, Ge et al. point out that solvents with good solubility can induce stronger molecular interaction compared to the thermal annealing treatment, thereby endowing molecules with better mobility to improve molecular crystallization and phase separation.^[^ ^9^, ^10^ ^]^ Accordingly, there is an urgent need for highly volatile and non‐halogenated solid additives, and further research is needed to effectively optimize molecular aggregation and stacking with the assistance of post‐treatment methods.

Interestingly, 1‐benzothiophene (BBT) with structure similar to the segment of active layer molecules has conjugated plane structure, which may induce ordered molecular stacking of active layer components. Moreover, BBT can be easily removed without high‐temperature thermal annealing treatment. Herein, we employed non‐halogenated and highly volatile BBT as solid additive combined with SVA post‐treatment to control morphology of active layer in OSCs. The synergistic effect of solid additive BBT and SVA on the performance of OSCs has been systematically investigated. Solid additive BBT can effectively regulate molecular aggregation of active layer (especially for acceptor Y6) and then SVA treatment can further fine‐tune the molecular stacking property. Compared with as‐cast devices, the BBT and SVA‐treated devices possess more suitable domain size and phase separation, enhanced molecular crystallinity, and tighter molecular packing, resulting in more effective exciton dissociation, faster and more balanced carrier transport, and suppressed charge recombination. The resultant devices exhibit enhanced short‐circuit current density (*J*_sc_) and fill factor (FF), leading to improved PCE. Ultimately, the efficiency of PM6:Y6 OSCs has been significantly improved from 15.58% to 17.53% by combining solid additive BBT and SVA treatment, and then further increased to 18.01% by using 2PACz as anode interface layer. Meanwhile, this joint strategy has good universality and has been successfully applied to the D18‐Cl:N3‐based OSCs. Accordingly, a high PCE of 19.53% was obtained, which is one of the highest reported PCE for binary OSCs to date. This work demonstrates that the combination of highly volatile solid additives and SVA treatment is an effective and simple morphology control strategy, which eliminates the dependence of volatile solid additives on high temperature when optimizing the morphology of active layer through volatile solid additives.

## 2 Results and Discussion

**Figure 1:**
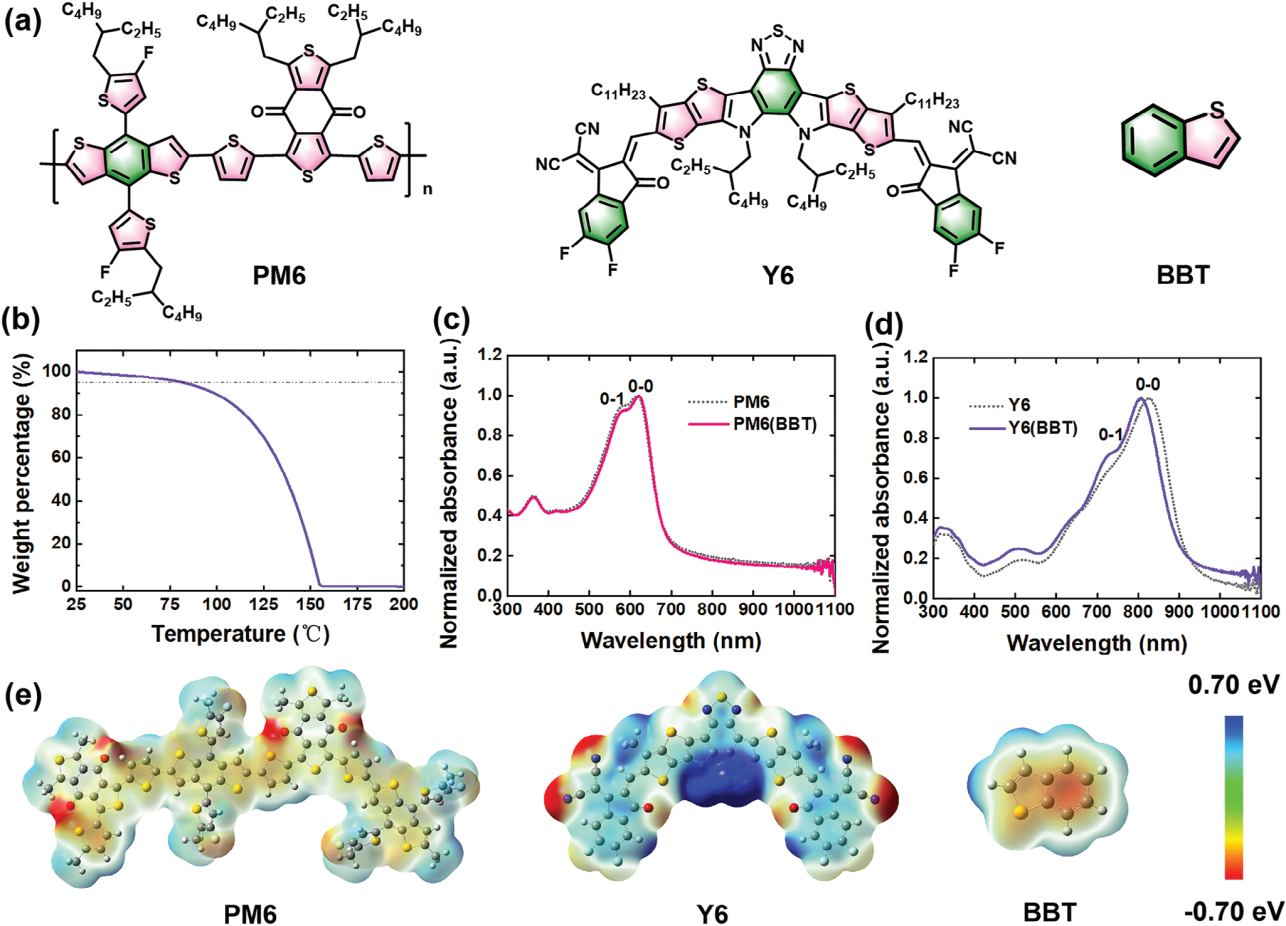
a) Chemical structures of donor PM6, acceptor Y6, and solid additive BBT. b) TGA plot of BBT at a scan rate of 5 °C min^−1^ under a nitrogen atmosphere. UV–vis spectra of c) PM6 neat film and d) Y6 neat film with and without BBT treatment. e) ESP distribution of PM6 dimer, Y6 and BBT.

The chemical structures of donor PM6, acceptor Y6, and solid additive BBT are shown in **Figure** **1**a. The solid additive BBT is simply composed of a benzene ring and thiophene, which are the most common repeating units in both donor PM6 and acceptor Y6. To verify the volatilization characteristics of solid additive BBT, thermogravimetric analysis (TGA) was first measured. As depicted in Figure 1b, the solid additive BBT exhibits a *T*_d_ (5% weight loss) at 80 °C, and completely disappears when heated to 155 °C, indicating BBT can be easily volatilized at low temperature. Additionally, the BBT solution was directly spin‐coated on the silicon wafer. According to Figure S1 (Supporting Information), there is no additive BBT residue on the silicon wafer after spin‐coating, suggesting it can be completely removed accompanying solvent volatilization during the spin‐coating process. Moreover, Fourier transform infrared spectroscopy (FT‐IR) of Y6 neat film with and without BBT additive and BBT powder was measured. As shown in Figure S2 (Supporting Information), the Y6 and Y6(BBT) films display similar FT‐IR spectra, which do not have the characteristic peak of BBT at 1054, 1013, 938, and 760 cm^−1^, etc. This result confirms that there is no residual BBT in the Y6(BBT) neat film.

Subsequently, UV–vis absorption spectra of polymer donor PM6 and non‐fullerene Y6 with/without BBT treatment were measured to probe the effects of additives on molecular aggregation property. As shown in Figure 1c,d, a slightly larger ratio of 0−0 and 0−1 vibronic peak intensity (*I*_0‐0_/*I*_0‐1_) in absorption is observed for the BBT‐treated films compared to the as‐cast PM6 neat film, suggesting stronger molecular aggregation of PM6 after introducing solid additive BBT.^[^ ^11^ ^]^ Conversely, the Y6 film exhibits a significantly lower ratio of *I*_0‐0_/*I*_0‐1_ and blue‐shifted absorption spectra after BBT treatment, indicating the preferred *H*‐aggregation.^[^ ^12^ ^]^ We also investigated the effect of additive BBT on the surface morphology of donor PM6 and acceptor Y6. As depicted in Figure S3 (Supporting Information), polymer donor PM6 has a clear nanofiber structure, of which the root‐mean‐square roughness (*R*_q_) slightly increases from 1.25 to 1.41 nm with the introduction of additive BBT. However, Y6 neat film has a uniform and smooth surface texture. After introducing the additive BBT, clustered spots are observed on the surface of Y6, and its *R*_q_ value increases from 0.99 to 1.25 nm. These results indicate that additive BBT can effectively regulate the molecular aggregation of active layer, especially for acceptor Y6.

To better analyze the intermolecular interactions between solid additive and active layer components, their electrostatic potential (ESP) distributions were calculated by density functional theory (DFT) at B3LYP/6‐311G(d,p) level (Taking into account the weak interaction, the dispersion correction is computed using Grimme's D3(BJ) method).^[^ ^8^, ^11^ ^]^ As shown in Figure 1e, the conjugated main chains of donor PM6 are mainly negative with positive centers at alkyl side chains. On the contrary, the backbone of acceptor Y6 is mostly positive with a negative region at cyano group moiety. It can be clearly observed that solid additive BBT has a negative iso‐surface. It can speculate that BBT can potentially interact with the acceptor Y6 and affect their molecular aggregation during the film‐formation process. Moreover, the non‐covalent interactions between acceptor Y6 and solid additive BBT were further analyzed by calculating the binding energy (*E*_b_) of them (Figure S4, Supporting Information). It is observed that BBT tends to interact with dithienothiophen[3,2‐*b*]‐pyrrolobenzothiadiazole (BTP) core unit of Y6 due to the large *E*_b_, thus regulating the aggregation state of the acceptor Y6 during the film‐forming process.

**Figure 2:**
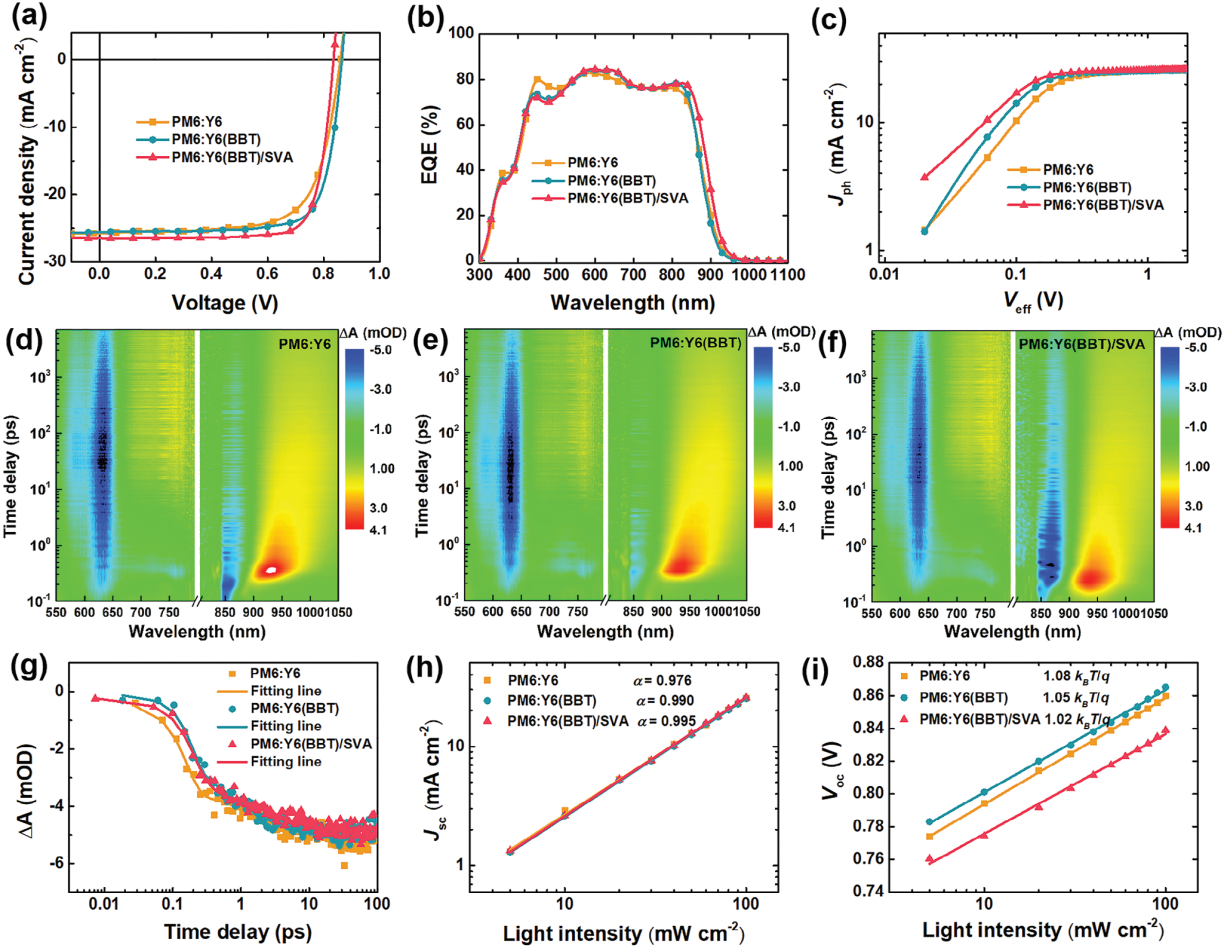
a) Optimized *J*–*V* curves and b) EQE spectra for the PM6:Y6, PM6:Y6(BBT), and PM6:Y6(BBT)/SVA OSCs. c) *J*_ph_ versus *V*_eff_ plots of the devices. The 2D color plots of TA spectra of d) PM6:Y6, e) PM6:Y6(BBT), and f) PM6:Y6(BBT)/SAV blend films with pump at 750 nm. g) TA traces probed at 636 nm recorded from PM6:Y6, PM6:Y6(BBT), and PM6:Y6(BBT)/SAV blend films. h) *J*_sc_ and i) *V*_oc_ versus light intensity plots of the devices.

To explore the effect of solid additive BBT on the performance of the devices, we fabricated the conventional OSCs with a configuration of indium tin oxide (ITO)/Anode interface layer (AIL)/Active layer/Cathode interface layer/Ag. The current density–voltage (*J–* *V*) curves of different devices are shown in **Figure** **2**a, and the corresponding detailed photovoltaic parameters are summarized in **Table** **1** and Tables S1 and S2 (Supporting Information). The as‐cast PM6:Y6 devices afford a PCE of 15.58%, with an open‐circuit voltage (*V*_oc_) of 0.860 V, a short‐circuit current density (*J*_sc_) of 25.73 mA cm^−2^, and an FF of 70.4%. After introducing the additive BBT, the PM6:Y6(BBT) devices deliver an enhanced *V*_oc_ of 0.865 V and FF of 77.0%, a *J*_sc_ of 25.57 mA cm^−2^, resulting in an improved PCE of 17.04%. Subsequently, SVA treatment was further applied to fine‐tune the morphology of the active layer, thus improving the performance of OSCs. After SVA post‐treatment, the PM6:Y6(BBT)/SVA devices display an enhanced *J*_sc_ of 26.50 mA cm^−2^ and FF of 79.2%, a decreased *V*_oc_ of 0.835 V, leading to a higher PCE of 17.53%. Furthermore, the PM6:Y6/SVA (without introducing additive BBT) devices afford a lower PCE of 16.68% compared to the PM6:Y6(BBT)/SVA devices. These results indicate that the combination of solid additive BBT and SVA post‐treatment can synergistically improve performance of OSCs. When the 2PACz was introduced as AIL (Figure S5, Supporting Information), a champion PCE of 18.01% was obtained for the PM6:Y6(BBT)/SVA devices, with a *V*_oc_ of 0.845 V, a *J*_sc_ of 27.01 mA cm^−2^, and an FF of 78.9% (Figure S6, Supporting Information), which is one of the highest reported PCE for the binary PM6:Y6 system to date.^[^ ^8e^ ^]^

**Table 1:** Detailed Photovoltaic Parameters for PM6:Y6‐based OSCs.

Active layer	*V*_oc_ [V]	*J*_sc_ [mA cm^−2^]	FF [%]	PCE [%]^a)^
Control	0.860	25.73 (24.58)^b)^	70.4	15.58 (15.30 ± 0.24)
PM6:Y6(BBT)	0.865	25.57 (24.54)	77.0	17.04 (16.88 ± 0.11)
PM6:Y6(BBT)/SVA	0.835	26.50 (25.26)	79.2	17.53 (17.22 ± 0.17)
PM6:Y6(BBT)/SVA^c)^	0.845	27.01 (25.79)	78.9	18.01 (17.66 ± 0.19)

^a)^ Average values and standard deviation were obtained from 12 individual devices;

^b)^ Integrated current density calculated from EQE spectra;

^c)^ The devices prepared with 2PACz as anode interface layer (AIL).

Furthermore, the external quantum efficiency (EQE) spectra of different devices were conducted and shown in Figure 2b. All samples present high EQE peaks in the range of 400–900 nm. Compared with PM6:Y6, the PM6:Y6(BBT) OSCs display slightly blue‐shifted EQE spectra, which is consistent with the changes in absorption spectra (Figure 1d; Figure S7, Supporting Information). After SVA treatment, the PM6:Y6(BBT)/SVA OSCs show a broader EQE spectra, which leads to enhanced *J*_sc_. Additionally, the integrated current density values for PM6:Y6, PM6:Y6(BBT) and PM6:Y6(BBT)/SVA devices are 24.58, 24.54, and 25.26 mA cm^−2^, respectively, which agree well with the *J*_sc_ values obtained from *J–* *V* curves with a deviation of <5%.

Meanwhile, exciton dissociation and charge transport processes were systematically studied in different devices. First, the photocurrent density (*J*_ph_ = *J*_L_–*J*_D_, where *J*_D_ and *J*_L_ are the current density of devices under dark and illumination conditions, respectively) versus the effective voltage (*V*_eff_ = *V*_0_–*V*_appl_, where *V*_appl_ is the applied voltage, and *V*_0_ is the voltage at *J*_ph_ = 0) was plotted to study the exciton dissociation and charge collection process in different devices.^[^ ^13^ ^]^ The exciton dissociation (*P*_diss_) and charge collection probability (*P*_coll_) can be determined by the ratio of *J*_ph_/*J*_sat_ (where *J*_sat_ is the saturation current density at a high *V*_eff_) under short‐circuit and maximum power output conditions, respectively. As shown in Figure 2c and Table S3 (Supporting Information), the PM6:Y6(BBT)‐based devices exhibit higher *P*_diss_ and *P*_coll_ values (98.3% and 89.6%) compared to PM6:Y6 (97.9% and 84.0%), suggesting that the introduction of additive BBT can facilitate exciton dissociation and charge collection. After SVA treatment, the *P*_diss_ and *P*_coll_ values of PM6:Y6(BBT)/SVA devices can be further improved to 98.7% and 91.8%, respectively, which can account for the higher *J*_sc_.

Subsequently, the charge transfer dynamics were investigated by femtosecond (fs) transient absorption (TA) spectroscopy measurements. Figure S8 (Supporting Information) and Figure 2d–f exhibit the TA spectra for the neat Y6 and different PM6:Y6 blend films when the acceptor Y6 was selectively excited by using an excitation wavelength at 750 nm. First, the TA spectra of the neat Y6 films display an obvious ground state bleaching (GSB) signal at 875 nm and an excited‐state absorption (ESA) signal at 940 nm after photoexcitation. In the blend films, an additional bleach signal at 630 nm (near the absorption peak of donor PM6) appear after photoexcitation. Obviously, emergence of the bleach signal of donor PM6 is accompanied by decay of the GSB signal of Y6, which is induced by hole transfer from Y6 to PM6. Subsequently, an ESA signal at 765 nm is observed, which can be assigned to the charge‐separated (CS) state of free polarons.^[^ ^14^ ^]^ As shown in Figure 2g, the hole transfer kinetics was studied by analyzing the kinetics of bleach signal of donor PM6, which can be fitted by bi‐exponential function, including two lifetimes *τ*_1_ and*τ*_2_. The fast component *τ*_1_ and slow component*τ*_2_ represent the time of exciton dissociation at the D/A interface and exciton diffusion to the interface, respectively.^[^ ^15^ ^]^ Here, compared with PM6:Y6 (*τ*_1_ = 1.23 ps, *τ*_2_ = 10.60 ps) and PM6:Y6(BBT) (*τ*_1_ = 0.74 ps, *τ*_2_ = 4.57 ps), the PM6:Y6(BBT)/SVA blend film displays a shorter *τ*_1_ (0.12 ps) and *τ*_2_ (3.51 ps), respectively. This result indicates that the synergistic effect of solid additive BBT and SVA treatment can facilitate faster exciton dissociation and exciton diffusion, leading to a higher *J*_sc_.

Next, the space‐charge limited current (SCLC) method was employed to evaluate the electron and hole mobility (*µ*_e_ and *µ*_h_).^[^ ^16^ ^]^ The results for electron‐only and hole‐only devices are presented in Figure S9 (Supporting Information) and the calculated *µ*_e_ and *µ*_h_ values are summarized in Table S4 (Supporting Information). The PM6:Y6‐based devices show a *µ*_h_ of 0.70 × 10^−4^ cm^2^ V^−1^ s^−1^ and a *µ*_e_ of 0.33 × 10^−4^ cm^2^ V^−1^ s^−1^. The *µ*_h_ of PM6:Y6(BBT) and PM6:Y6(BBT)/SVA‐based devices gradually increases to 1.15 × 10^−4^ and 1.76 × 10^−4^ cm^2^ V^−1^ s^−1^, while the *µ*_e_ of them correspondingly increases to 0.70 × 10^−4^ and 1.97 × 10^−4^ cm^2^ V^−1^ s^−1^. The faster and more balanced carrier transport can well explain the gradually increasing FF for the PM6:Y6, PM6:Y6(BBT), and PM6:Y6(BBT)/SVA‐based devices.^[^ ^17^ ^]^

Furthermore, the charge recombination properties were investigated by analyzing the relationship between *J*_sc_, *V*_oc,_ and light intensity (*P*_light_). The dependence of *J*_sc_ on light intensity (*P*_light_) can reflect bimolecular recombination in devices. The correlation between *J*_sc_ and *P*_light_ obeys the power law *J*_sc_ ∝ (*P*_light_)^α^.^[^ ^18^ ^]^ As shown in Figure 2h, the PM6:Y6(BBT) and PM6:Y6(BBT)/SVA‐based devices possess higher α values of 0.990 and 0.995 compared to PM6:Y6(0.976), indicating decreased bimolecular recombination in devices. Moreover, the dependence of *V*_oc_ on *P*_light_ follows *V*_oc_ ∝ (*nk*_B_ *T*/*q*) ln(*P*_light_), where *T*, *k*_B_, and *q* are the absolute temperature, Boltzmann constant, and elementary charge, respectively.^[^ ^19^ ^]^ As exhibited in Figure 2i, the slope of PM6:Y6, PM6:Y6(BBT) and PM6:Y6(BBT)/SVA‐based devices are 1.08 *k*_B_ *T*/*q*, 1.05 *k*_B_ *T*/*q* and 1.02 *k*_B_ *T*/*q*, respectively, indicating that the trap‐assisted recombination was also suppressed in PM6:Y6(BBT) and PM6:Y6(BBT)/SVA‐based devices. These results suggest that the combination of solid additive BBT and SVA treatment can effectively inhibit bimolecular recombination and trap‐assisted recombination in devices, which agrees well with the increased *J*_sc_ and FF.

**Figure 3:**
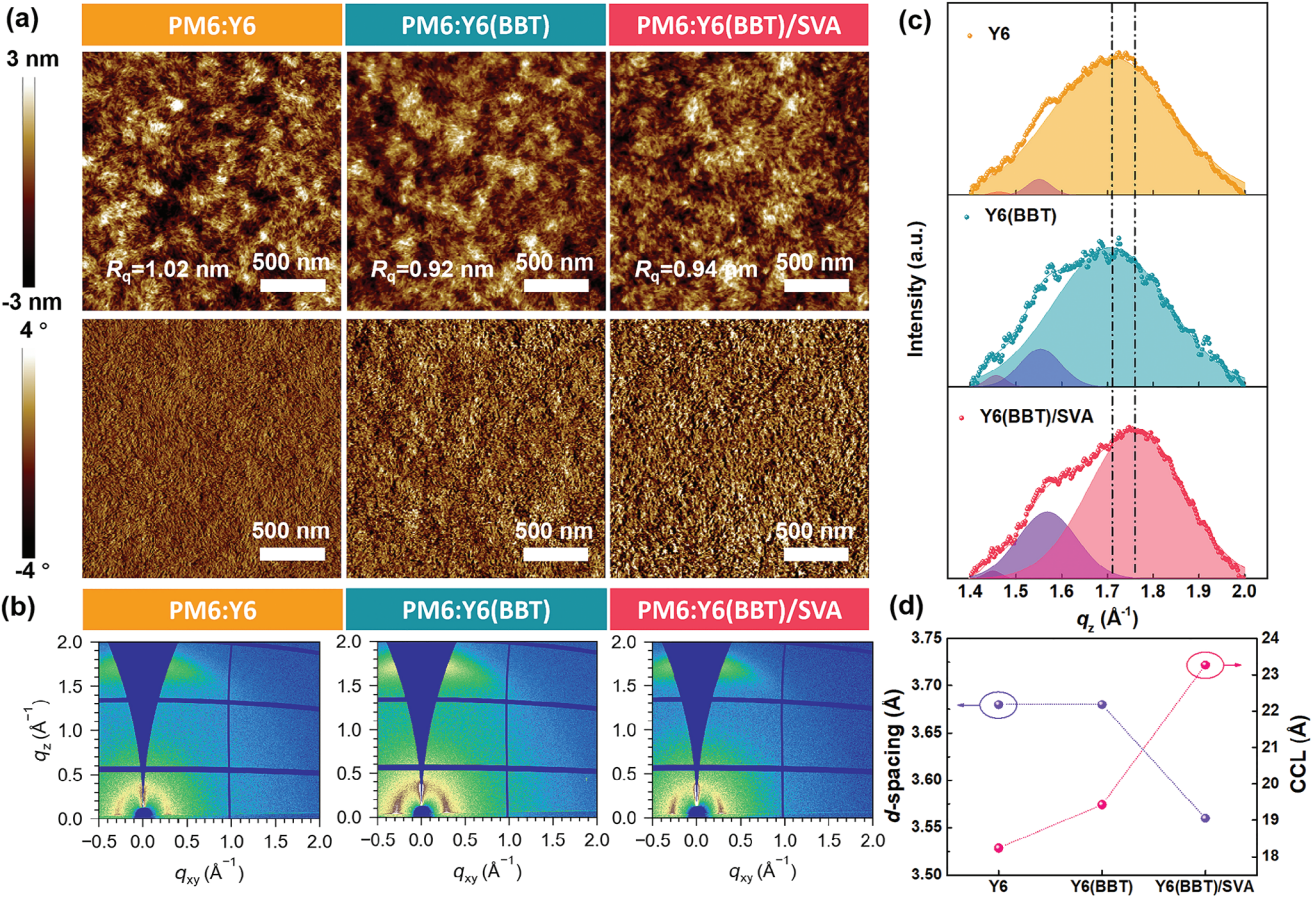
a) AFM height images and phase images of PM6:Y6, PM6:Y6(BBT), and PM6:Y6(BBT)/SVA blend films. b) 2D GIWAXS patterns of PM6:Y6, PM6:Y6(BBT), and PM6:Y6(BBT)/SVA blend films. c) *π*–*π* stacking peak of Y6, Y6(BBT), and Y6(BBT)/SVA neat films in the OOP direction. d) *d*‐spacing and CCL values of *π*–*π* stacking peak for Y6, Y6(BBT), and Y6(BBT)/SVA neat films.

The surface morphology of the blend films was observed by atomic force microscopy (AFM) measurement. The corresponding height and phase images are shown in **Figure** **3**a. All the blend films exhibit an excellent nanofiber interpenetrating network. Compared with as‐cast PM6:Y6, the PM6:Y6(BBT) and PM6:Y6(BBT)/SVA blend films have a slightly lower *R*_q_ and more obvious phase separation, indicating that the solid additive BBT and SVA treatment can synergistically regulate the molecular aggregation and phase separation of the active layer. The optimized morphology can promote free charge generation, facilitate charge transport, and reduce charge recombination, leading to enhanced *J*_sc_ and FF.

Also, grazing‐incidence wide‐angle X‐ray scattering (GIWAXS) measurements were performed to analyze the molecular stacking of neat and blend films (Figure S10, Supporting Information; Figure 3b). The 2D GIWAXS patterns and the corresponding line cuts of PM6 and Y6 neat films are presented in Figures S10 and S11a (Supporting Information), respectively. The detailed information of (010) *π*–*π* stacking peak is summarized in Table S5 (Supporting Information). The PM6 neat film exhibits an obvious (010) *π*–*π* stacking peak in the out‐of‐plane (OOP) direction and a strong (100) lamellar peak in the in‐plane (IP) direction, indicating a dominant face‐on orientation. Meanwhile, the Y6 neat film, which possesses a (010) *π*–*π* stacking peak in OOP direction (at *q* = 1.71 Å^−1^) and (100) lamellar peak in the IP direction, also predominantly adopts a face‐on orientation.^[^ ^1f^ ^]^ Given that additive BBT is more inclined to interact with the acceptor Y6, the molecular stacking characteristics of Y6 neat film with different treatment conditions were also investigated. As shown in Figure 3c,d, the peak location of *π*–*π* stacking for the Y6 neat films is not shifted (at *q* = 1.71 Å^−1^) but the corresponding crystal coherence length (CCL) value increases from 18.24 to 19.43 Å after introducing additive BBT, suggesting enhanced molecular crystallinity. However, after SVA treatment, the *π*–*π* stacking peak shifts from 1.71 (*d* = 3.68 Å) to 1.76 Å^−1^ (*d* = 3.56 Å), and the relevant CCL value further increases to 23.27 Å, indicating tighter molecular packing and increased molecular crystallinity.

As for the blend film, three samples prefer face‐on orientation with a (010) *π*–*π* stacking peak in the OOP direction and (100) lamellar peak in the IP direction, which favors charge transport in the vertical direction.^[^ ^4c^ ^]^ As shown in Figure 3b and Figure S11b,c (Supporting Information), the (010) *π*–*π* stacking peak of PM6:Y6 and PM6:Y6(BBT) blend films are located at *q* = 1.72 Å^−1^, corresponding to a *π–* *π* stacking distance of 3.66 Å. The PM6:Y6(BBT)/SVA blend film exhibits a slightly smaller *π*–*π* stacking distance 3.62 Å (*q* = 1.74 Å^−1^) after SVA treatment. Additionally, the CCL values of *π–* *π* stacking peak increase sequentially in the order of PM6:Y6 (19.77 Å), PM6:Y6(BBT) (21.26 Å), and PM6:Y6(BBT)/SVA (25.82 Å), suggesting that the molecular crystallinity is sequentially enhanced. These results indicate that the combined strategy of additive BBT and SVA treatment can effectively reduce *π*–*π* stacking distance and increase molecular crystallinity, which can facilitate charge transport and suppress charge recombination.

Additionally, grazing‐incidence small‐angle X‐ray scattering (GISAXS) measurements were performed to quantitatively analyze the domain features in blend films. The 2D GISAXS patterns and the corresponding 1D GISAXS profiles along in‐plane direction of PM6:Y6 blend films are exhibited in Figure S12 (Supporting Information), which can be fitted by using the Debye–Anderson–Brumberger (DAB) model and fractal‐like network model,^[^ ^20^ ^]^ and the detailed fitting data were summarized in Table S6 (Supporting Information). According to previous works, 2*R*_g_ can be regarded as the domain size of acceptor phase.^[^ ^21^ ^]^ The 2*R*_g_ value of PM6:Y6(BBT) and PM6:Y6(BBT)/SVA blend films are 29.8 and 30.4 nm, respectively, which is smaller than that of PM6:Y6 (33.9 nm). This result implies that the combined strategy can appropriately reduce domain size of acceptor and regulate phase separation of blend films, which can shorten exciton diffusion distance and facilitate exciton dissociation. As discussed above in GIWAXS and GISAXS measurements, the optimized PM6:Y6(BBT)/SVA blend film exhibits increased molecular crystallinity, tighter molecular packing, more suitable domain size and phase separation compared to the as‐cast PM6:Y6 film, leading to efficient exciton dissociation and charge transport. This can well account for the improved *J*_sc_ and FF.

**Figure 4:**
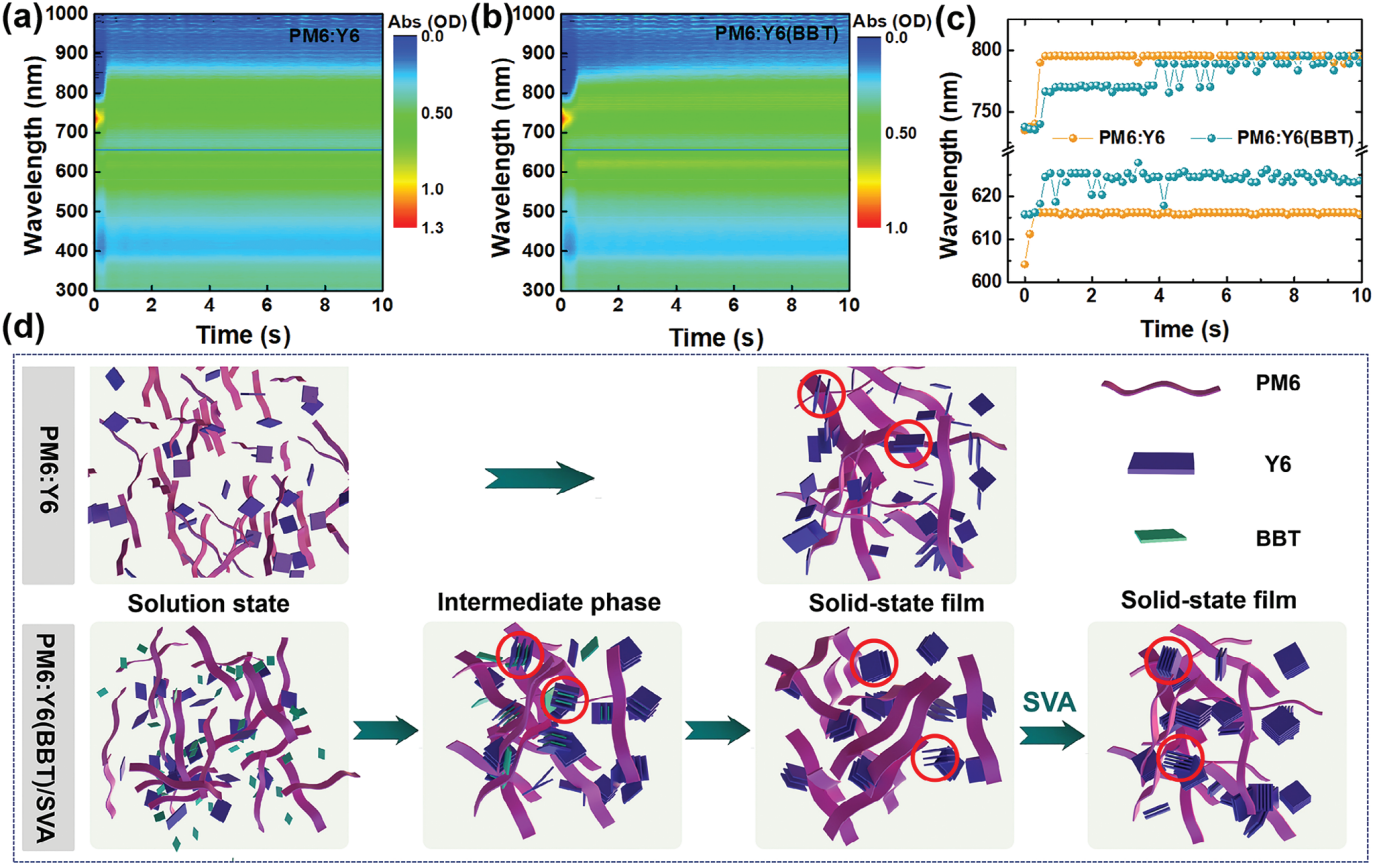
Contour maps of in situ UV–vis absorption spectra for a) PM6:Y6 and b) PM6:Y6(BBT) blend films. c) Time evolution of peak position of donor and acceptor in PM6:Y6 blend films with and without BBT treatment. d) Schematic diagram of the synergistic effect of solid additive BBT and SVA on film formation.

To better understand the effect of additive BBT on the film‐forming process of the active layer, in situ absorption was performed. The in situ absorption spectra of the samples with and without additive BBT treatment are depicted in **Figure** **4**a–c.^[^ ^22^ ^]^ Time‐resolved peak positions and intensities of donor and acceptor can effectively reveal the process of film formation. In summary, the film formation process of the active layer can be divided into three stages.^[^ ^8b^ ^]^ In stage i, droplets of solution drop onto the substrate. Under centrifugal force, the precursor solution gradually flows out and fills the substrate. During this process, the peak intensity of the donor and acceptor rapidly decreases, while the peak position changes relatively little. In stage ii, the solution gradually transforms into a solid thin film. The peak position of the donors and acceptors and the absorption spectra (Figure S13, Supporting Information) gradually show a bathochromic shift, indicating enhanced molecular aggregation in the thin film. In stage iii, the all‐solid film has been formed, and the peak intensity and position of the donor and acceptor remain stable. Intriguingly, unlike the untreated process, a momentary intermediate phase that donor PM6 and non‐fullerene acceptor Y6 mixed well with additive BBT is formed during stages ii and iii after introducing additive BBT, which only lasts for ≈5 s. Subsequently, the additive BBT rapidly evaporates with the rotation of the substrate, which provides sufficient room for the assembly of donor and acceptor.

As discussed above, the schematic diagram of the film‐formation process of the active layer is shown in Figure 4d. After introducing solid additive BBT, additive BBT may prefer to interact with acceptor due to the electrostatic interaction. During the film‐formation process, a short‐lived intermediate phase is formed, which delays the film‐formation process, thereby regulating the molecular aggregation characteristics and facilitating more ordered molecular stacking in the solid film. Subsequently, the SVA treatment provides a solvent atmosphere and momentum for molecular rearrangement, resulting in more ordered and tighter molecular stacking.

**Figure 5:**
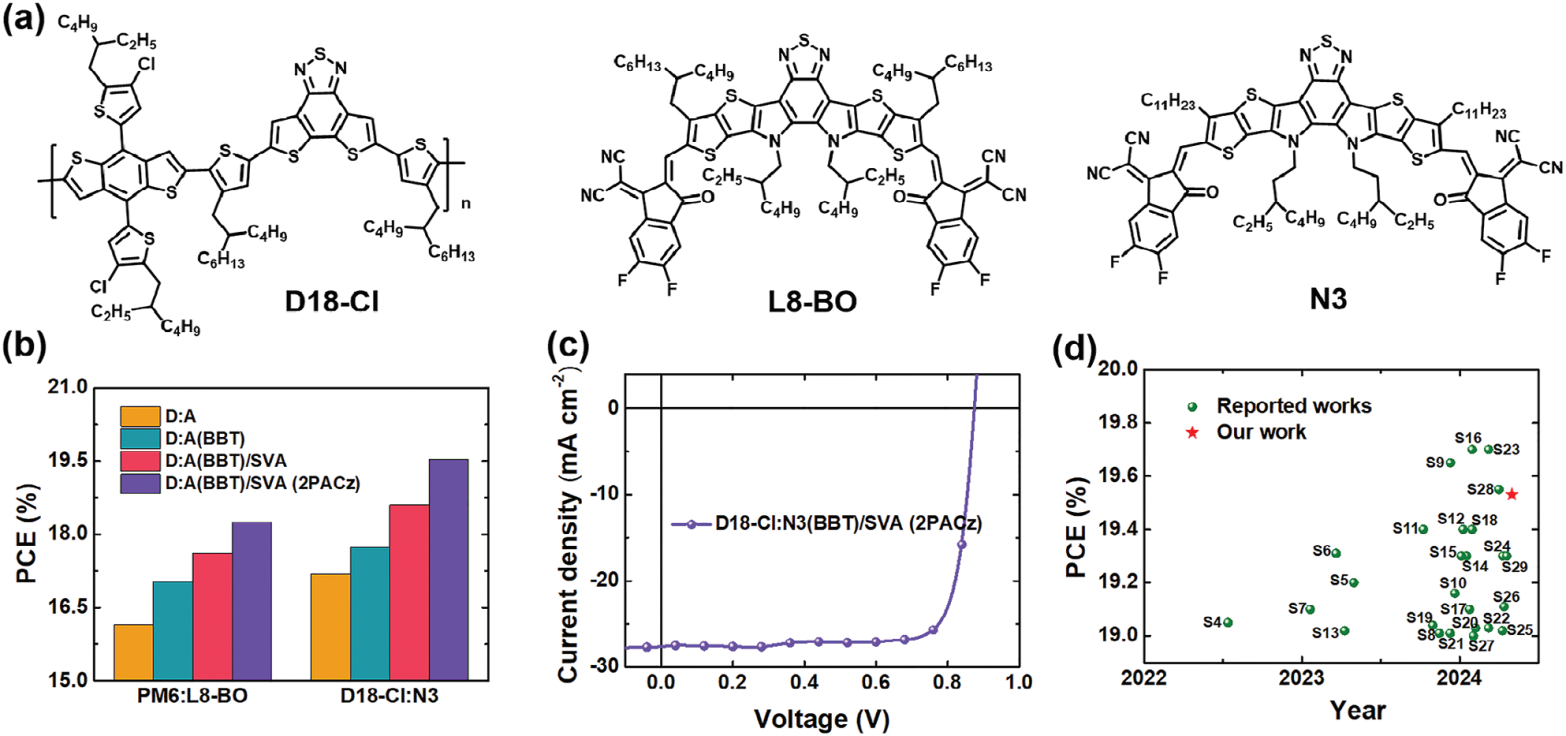
a) Chemical structures of donor D18‐Cl, acceptors L8‐BO and N3. b) Histogram of champion PCE from PM6:L8‐BO and D18‐Cl:N3 OSCs under different conditions. c) *J–* *V* curve of D18‐Cl:N3(BBT)/SVA devices by using 2PACz as AIL. d) Summary of recently reported binary OSCs with efficiency exceeding 19%.

In addition, this combined strategy was also applied to other binary OSCs systems to verify its universality, including PM6:L8‐BO and D18‐Cl:N3 (**Figure** **5**a). The corresponding *J–* *V* curves and EQE spectra are shown in Figure S14 (Supporting Information), and the detailed photovoltaic parameters are summarized in **Table** **2** and Figure 5b. Compared with as‐cast PM6:L8‐BO devices (16.16%), the PM6:L8‐BO(BBT)/SVA devices exhibit a higher PCE of 17.62% due to its significantly enhanced *J*_sc_ and FF. As expected, the efficiency of PM6:L8‐BO(BBT)/SVA devices is further improved to 18.25% by using 2PACz interface layer. Notably, the PCE of D18‐Cl:N3 devices has remarkably increased from 17.18% to 18.60% after solid additive BBT and SVA treatment. Undoubtedly, when using 2PACz as AIL, the D18‐Cl:N3(BBT)/SVA devices deliver a champion PCE of 19.53%, with a *V*_oc_ of 0.875 V, a *J*_sc_ of 27.63 mA cm^−2^ and an FF of 80.8% (Figure 5c). According to statistics (Figure 5d and Table S7, Supporting Information), the 19.53% efficiency is one of the highest reported efficiencies for binary OSCs to date. These results indicate that the combination of solid additive BBT and SVA treatment is an effective method to improve performance of OSCs, which also has good universality.

**Table 2:** Summary of Photovoltaic Parameters for PM6:L8‐BO and D18‐Cl:N3‐based OSCs.

Active layer		*V*_oc_ [V]	*J*_sc_ [mA cm^−2^]	FF [%]	PCE [%]^a)^
PM6:L8‐BO	Control	0.912	24.76 (23.54)^b)^	71.6	16.16 (15.83 ± 0.24)
	D:A(BBT)	0.896	24.30 (23.36)	78.2	17.03 (16.80 ± 0.20)
	D:A(BBT)/SVA	0.884	25.01 (23.98)	79.6	17.62 (17.24 ± 0.26)
	D:A(BBT)/SVA^c)^	0.881	26.20 (25.06)	79.0	18.25 (17.92 ± 0.26)
D18‐Cl:N3	Control	0.872	26.31 (25.30)	74.9	17.18 (17.00 ± 0.13)
	D:A(BBT)	0.912	24.43 (23.41)	79.6	17.74 (17.41 ± 0.27)
	D:A(BBT)/SVA	0.870	26.71 (25.83)	80.1	18.60 (18.33 ± 0.21)
	D:A(BBT)/SVA^c)^	0.875	27.63 (26.48)	80.8	19.53 (19.24 ± 0.22)

^a)^ Average values and standard deviation were obtained from 12 individual devices;

^b)^ Integrated current density calculated from EQE spectra;

^c)^ The devices prepared with 2PACz as AIL.

## 3 Conclusion

In summary, a non‐halogenated solid additive BBT was developed to control the morphology of active layer in OSCs. Unlike previously reported solid additives that require high‐temperature thermal annealing to be removed from the active layer, solid additive BBT can spontaneously volatilize during the film‐forming process. The film‐forming kinetics of the active layer were studied by in situ absorption spectra. The results indicate that a momentary intermediate phase where the donor, acceptor, and additive BBT mix well, forms initially and then quickly disappears as additive BBT volatilizes, thereby regulating the molecular aggregation. Subsequently, SVA treatment was used to further improve the molecular stacking. After solid additive BBT and SVA treatment, an ideal morphology with nanofiber interpenetrating network, suitable domain size and phase separation, increased molecular crystallinity, and tight molecular packing is obtained, which can promote exciton dissociation, facilitate charge transport, and suppress charge recombination, resulting in enhanced *J*_sc_ and FF. As a result, the PCE of PM6:Y6‐based OSCs was significantly improved from 15.58% to 17.53%, which can be further improved to 18.01% when the device was prepared with 2PACz as anode interface layer. Meanwhile, this joint strategy has good universality in other OSCs systems. In D18‐Cl:N3 system, a high PCE of 19.53% was obtained. To the best of our knowledge, it is one of the highest reported efficiencies for binary OSCs to date. This work demonstrates that the combination of highly volatile solid additive BBT and SVA treatment is a simple and effective morphology control method to improve the photovoltaic performance of OSCs, which provides new ideas for the preparation of high‐performance OSCs.

## Conflict of Interest

The authors declare no conflict of interest.

## Supporting information



Supporting Information

Supporting Information

## Data Availability

The data that support the findings of this study are available from the corresponding author upon reasonable request.
