# Supplementary material for: Gradual Optimization of Molecular Aggregation and Stacking Enables Over 19% Efficiency in Binary Organic Solar Cells
Source: Adv Sci (Weinh). 2024 Oct 2;11(44):2409867. doi: 10.1002/advs.202409867 (PMC11600276; doi:10.1002/advs.202409867)
Supplement: Supplementary file 1 — Supporting Information [file ADVS-11-2409867-s001.docx]

Supporting Information

**Gradual Optimization of Molecular Aggregation and Stacking Enables over 19% Efficiency in Binary Organic Solar Cells**

Jianqiang Qin, Linze Wu, Sihao Huang, Zeping Ou, Xiaowu Wang, Yingguo Yang,* Yujie Zheng, Kuan Sun, Zeyu Zhang,* Zhiping Hu, Zhengzheng Liu, Yuxin Leng, and Juan Du*

J. Qin, X. Wang, Z. Zhang, Z. Hu, Y. Leng, J. Du

School of Physics and Optoelectronic Engineering, Hangzhou Institute for Advanced Study, University of Chinese Academy of Sciences, Hangzhou 310024, China;

E-mail: zhangzeyu@ucas.ac.cn (Z. Zhang); dujuan@siom.ac.cn (J. Du)

L. Wu, S. Huang, Z. Liu, Y. Leng

State Key Laboratory of High Field Laser Physics and CAS Center for Excellence in Ultra-intense Laser Science, Shanghai Institute of Optics and Fine Mechanics (SIOM), Chinese Academy of Sciences (CAS), Shanghai 201800, China;

Z. Ou, Y. Zheng, K. Sun

MOE Key Laboratory of Low-grade Energy Utilization Technologies and Systems, School of Energy & Power Engineering, Chongqing University, Chongqing 400044, China;

Y. Yang

School of Microelectronics, Fudan University, Shanghai 200433, China;

E-mail: yangyingguo@fudan.edu.cn (Y. Yang)

**Supporting information**

**Experimental Section**

**1. Materials**

Polymer donor PM6 and non-fullerene acceptors (L8-BO and N3) were purchased from Derthon Optoelectronic Materials Science Technology Co., Ltd. Polymer donor D18-Cl was purchased from eFlexPV Limited. Non-fullerene acceptor Y6 and cathode interface materials (PDIN and PDINN) were purchased from Solarmer Material Inc. Solid additive 1-benzothiophene (BBT) was purchased from TCI CO., LTD. All the materials were directly used without any further purification.

**2. Device fabrication and measurement**

The OSCs devices were fabricated with a conventional structure of ITO/PEDOT:PSS or 2PACz/active layer/PDINN or PDIN/Ag. Firstly, the ITO substrates (15 Ω sq^-1^) were sequentially sonicated in detergent, deionized water, acetone, and isopropyl alcohol for 20 min, and then treated with UV-ozone for 15 min. Subsequently, a 30-nm-thick PEDOT:PSS (CLEVIOS^TM^ P VP AI 4083) film was deposited on the ITO substrate, and then annealed at 130 ℃ for 10 min in ambient air. For 2PCAz devices, the 2PCAz (0.5 mg mL^−1^ in isopropyl alcohol) was spin-coated on the ITO substrates at 4000 rpm for 30 s.^[^[^1^](#_ENREF_1)^]^ All the active layers were prepared in N_2_-filled glove box. The PM6:Y6 or PM6:L8-BO blend was dissolved in chloroform (CF) with a D:A ratio of 1:1.2 and a total concentration of 17.5 mg mL^-1^ with different BBT solid additive contents. However, the D18-Cl:N3 blend was dissolved in CF with a D:A ratio of 1:1.4 and a total concentration of 16 mg mL^-1^. The blend solutions were spin-coated at 3000 rpm for 30 s onto the PEDOT:PSS layer. The solvent vapor annealing (SVA) treatment was performed by putting the blend films immediately into an aluminum box with a volume of 50 mL, and then 80 uL of carbon disulfide (CS_2_) solvent was added around the blend films. The SVA processing time was further optimized by adjusting the placement time. Subsequently, a thin PDINN (2 mg mL^-1^ in methanol) or PDIN (2 mg mL^-1^ in methanol with 0.3 vol% acetic acid) layer was spin-coated on the top of active layer. Finally, a 100-nm-thick Ag electrode was thermally deposited under 2×10^-4^ Pa. The effective area of device was 0.0395 cm^2^. The *J*-*V* curves of devices were measured by using a Keysight 2901BL Source Meter under AM 1.5G (100 mW cm^-2^) generated from a solar simulator. The illumination intensity was determined by a silicon photodiode calibrated by the National Renewable Energy Laboratory (NREL). The external quantum efficiency (EQE) spectra were measured by using QE-R equipment system (Enli Tech).

**3. Ultraviolet-Visible (UV-Vis) absorption spectra, Fourier transform infrared (FT-IR) spectra and *in-situ* UV-Vis absorption spectra**

UV-Vis absorption spectra were performed by using a Cary 60 UV-Vis spectrophotometer (Agilent Technologies). FT-IR measurement was conducted by using a FT-IR spectrometer (Thermo Scientific Nicolet 6700). Y6 and Y6(BBT) films was prepared on BaF_2_ substrate via spin-coating method. FTIR spectrum of BBT powder was measured in the ATR mode. The *in-situ* UV-Vis absorption spectra were conducted by using an *in-situ* dynamic spectrometer DU-300.

**4. Transient absorption (TA) spectroscopy**

TA spectroscopy measurement was conducted on a TA spectrometer (Ultrafast Systems, Helios Fire). The fundamental output pulse from a Ti:sapphire regenerative ampliﬁer (800 nm, 35 fs, 1 kHz) was split into two beams. The pump beam was generated from an optical parametric ampliﬁer (In this work, the wavelength of pump pulse was tuned at 750 nm to selectively excite the acceptor.); Another beam was used to pump the calcium ﬂuoride (CaF_2_) crystal to generate the probe pulses. A mechanical chopper with a synchronized readout of a CMOS detector can capture the change in probe spectra, thus calculating the relative diﬀerential absorption (ΔA). The samples are prepared by spin-coating the neat and blend film solutions on the quartz substrates.

**5. SCLC measurement**

The hole mobility and electron mobility were measured via space-charge-limited current (SCLC) method. The hole-only devices were fabricated with architecture of ITO/PEDOT:PSS/active layer/MoO_3_/Ag, and the electron-only devices were fabricated with configuration of ITO/ZnO/active layer/PDINN/Ag.^[^[^2^](#_ENREF_2)^]^ The *J*-*V* curves were measured by using Keysight 2901BL Source Meter in the dark. The mobility was obtained by fitting the dark current with the following equation:

$$J=\frac{9}{8}\varepsilon_{0}\varepsilon_{r}\mu\frac{V^{2}}{L^{3}}$$

where *J* is the current density, *ε*_0_ is the permittivity of the vacuum, *ε*_γ_ is the dielectric constant of material, *μ* is zero-field mobility (*μ*_e_ and *μ*_h_ are electron mobility and hole mobility, respectively), *V* is the effective voltage (*V* = *V*_appl_ - *V*_bi_, where *V*_appl_ and *V*_bi_ are the applied voltage and built-in potential, respectively), *L* is the film thickness. The mobility can be obtained from the *J*^1/2^-*V* plot.^[^[^3^](#_ENREF_3)^]^

**6. TGA**

TGA was conducted on a METTLER TOLEDO TGA/DSC 3+ thermogravimetric analyzer in the range of 25 to 250 ℃ with a heating rate of 5 ℃ min^−1^ under a nitrogen atmosphere.

**7. Morphology characterization**

Atomic force microscope (AFM) measurements were performed on a Dimension Icon (Bruker) by using in tapping mode. GIWAXS and GISAXS measurements were conducted using the beamline BL02U2 and BL19U2 at the Shanghai Synchrotron Radiation Facility (China), respectively. All the samples were prepared on Si substrate using optimized conditions for devices. The incident angle was set to 0.2°. The crystal coherence length (CCL) can be calculated by using the Scherrer equation:^[^[^4^](#_ENREF_4)^]^

$$\text{CCL=}\frac{2\pi K}{\Delta q}$$

Where *K* and Δ*q* are the shape factor (*K* = 0.9) and the full-width at half-maximum (FWHM) of the diffraction peak, respectively.

**Supporting Figures**


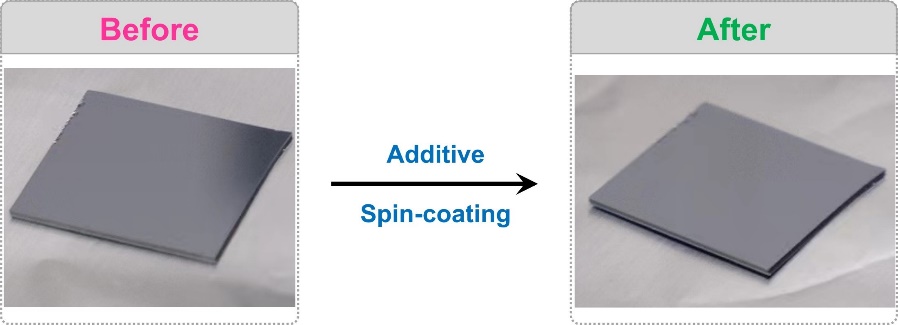


**Figure S1** The photograph of BBT solution before and after spin-coating on silicon wafer.


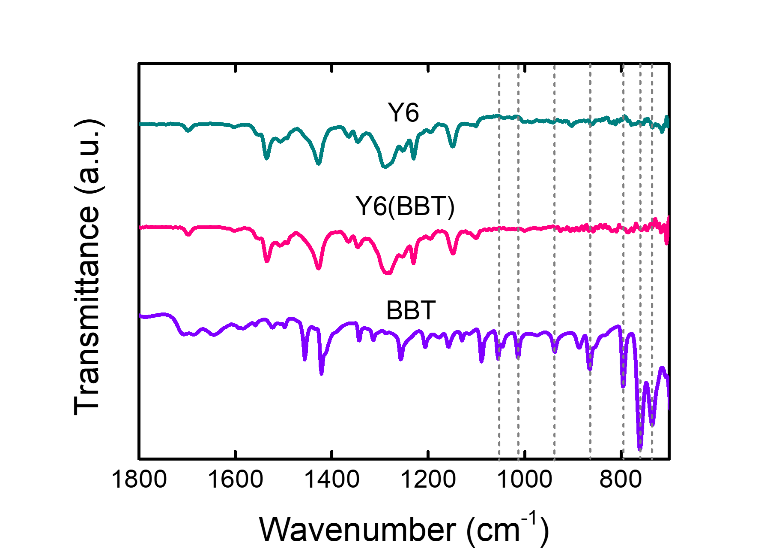


**Figure S2** FT-IR spectra of BBT powder, Y6 and Y6(BBT) film.


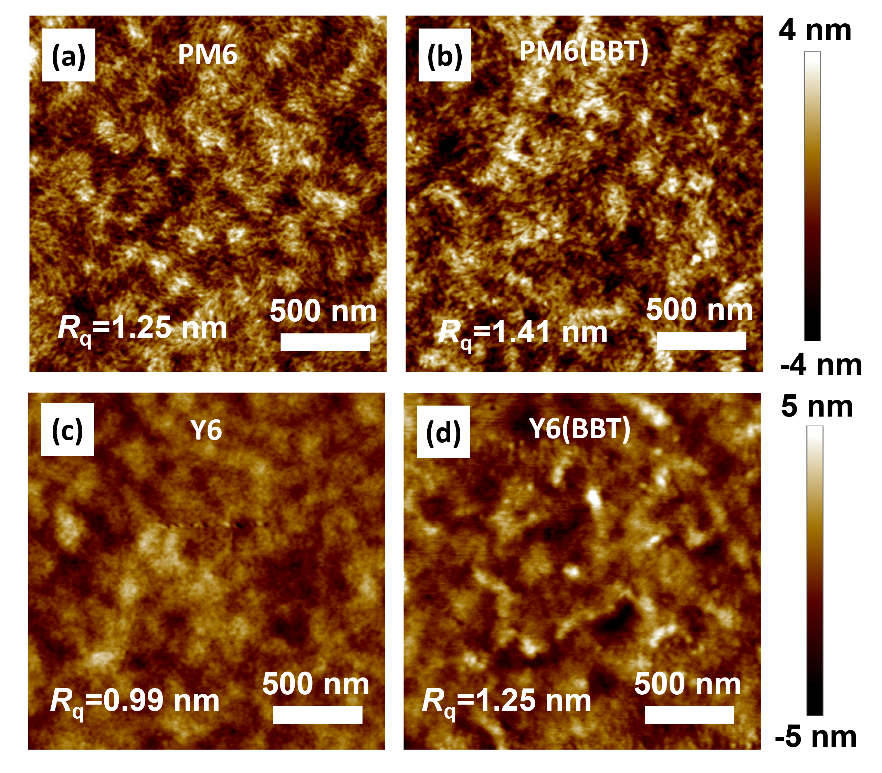


**Figure S3** The AFM height images for (a) PM6, (b) PM6(BBT), (c) Y6, and (d) Y6(BBT) neat films.


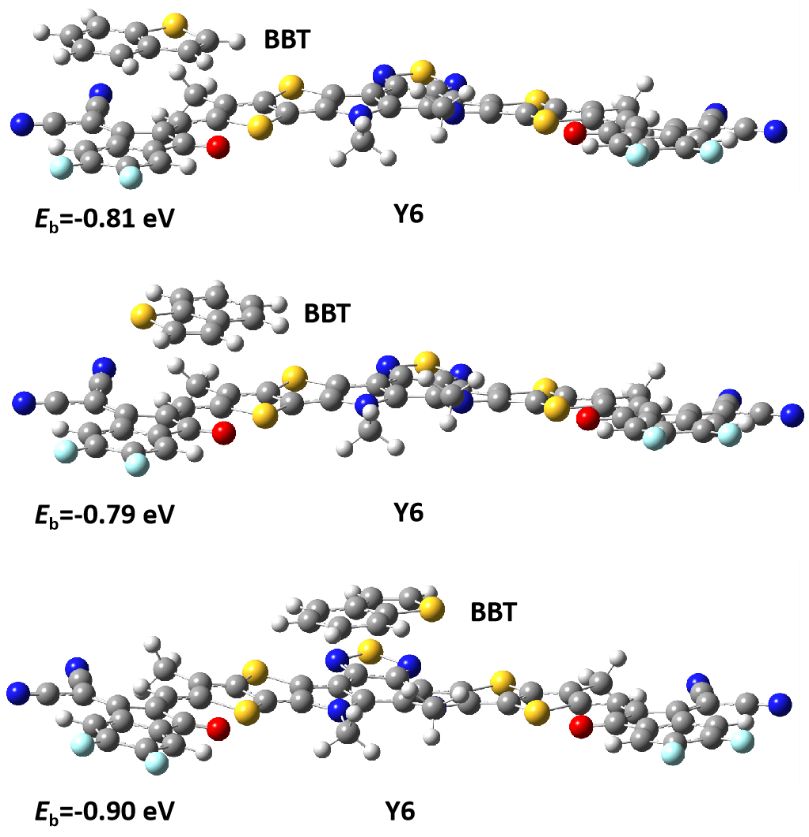


**Figure S4** The binding energy between the optimized geometry of Y6 and BBT calculated from DFT.


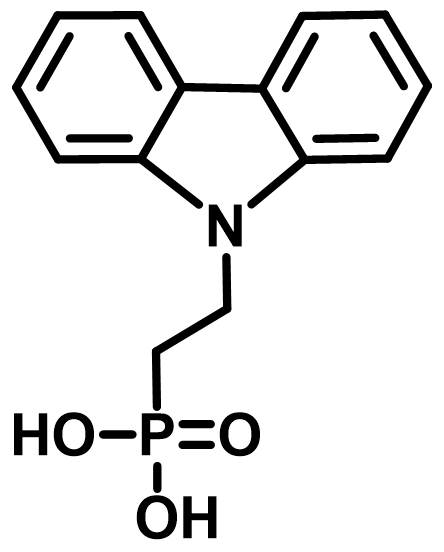


**Figure S5** Chemical structures of 2PACz.


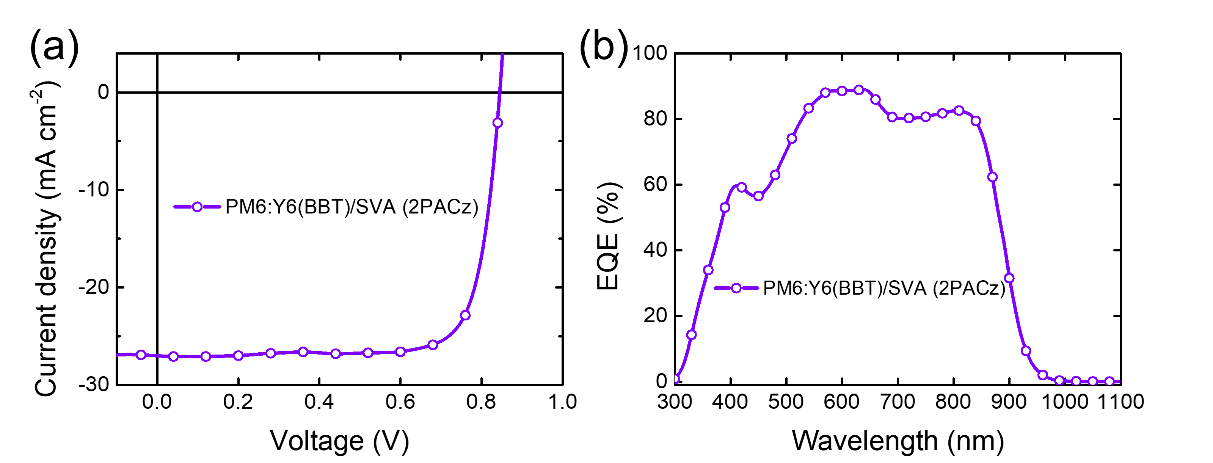


**Figure S6** (a) *J*-*V* curve and (b) corresponding EQE spectrum of PM6:Y6(BBT)/SVA OSCs prepared with 2PACz as AIL.


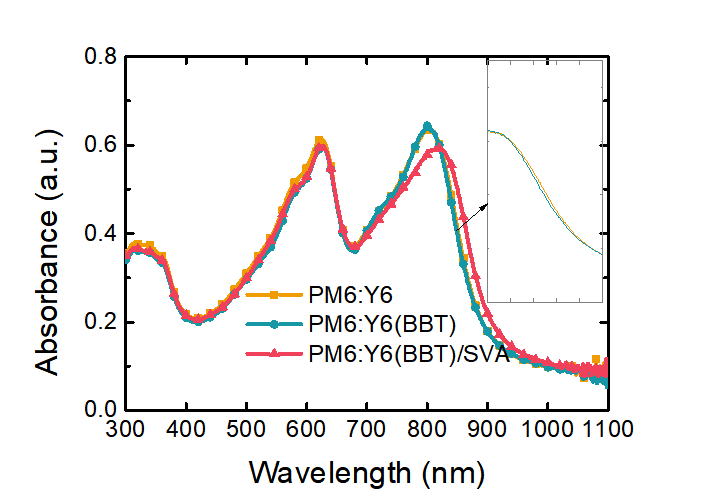


**Figure S7** The UV-Vis absorption spectra of PM6:Y6, PM6:Y6(BBT), and PM6:Y6(BBT)/SVA blend films. The inset shows the zoomed-in UV-Vis spectra.


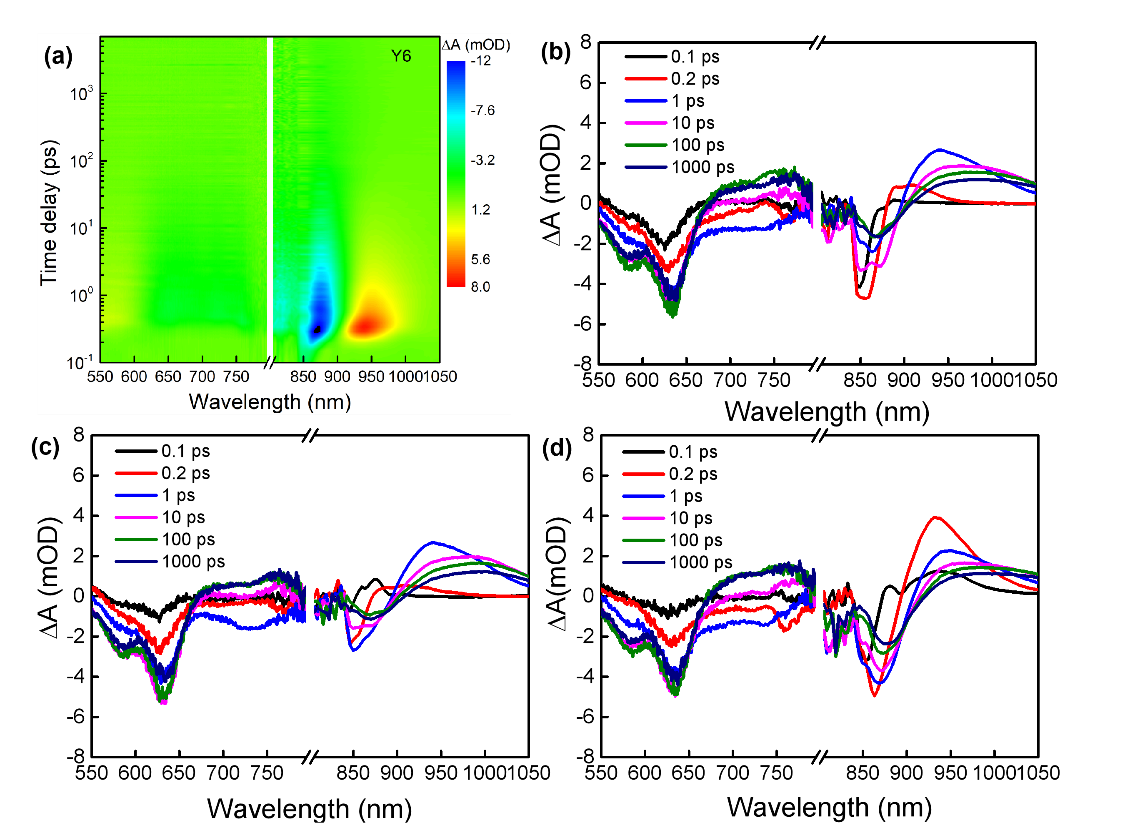


**Figure S8** (a) The 2D color plots of TA spectra for the Y6 neat films with pump at 750 nm. The TA spectra of (b) PM6:Y6, (c) PM6:Y6(BBT), and (d) PM6:Y6(BBT)/SAV blend films at different time delay.


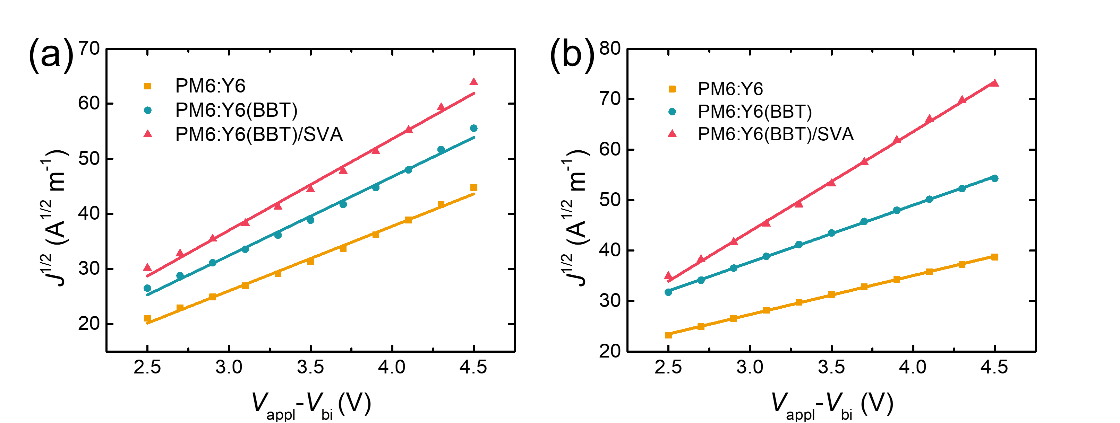


**Figure S9** The *J*^1/2^-*V* plots for (a) hole-only devices and (b) electron-only devices.


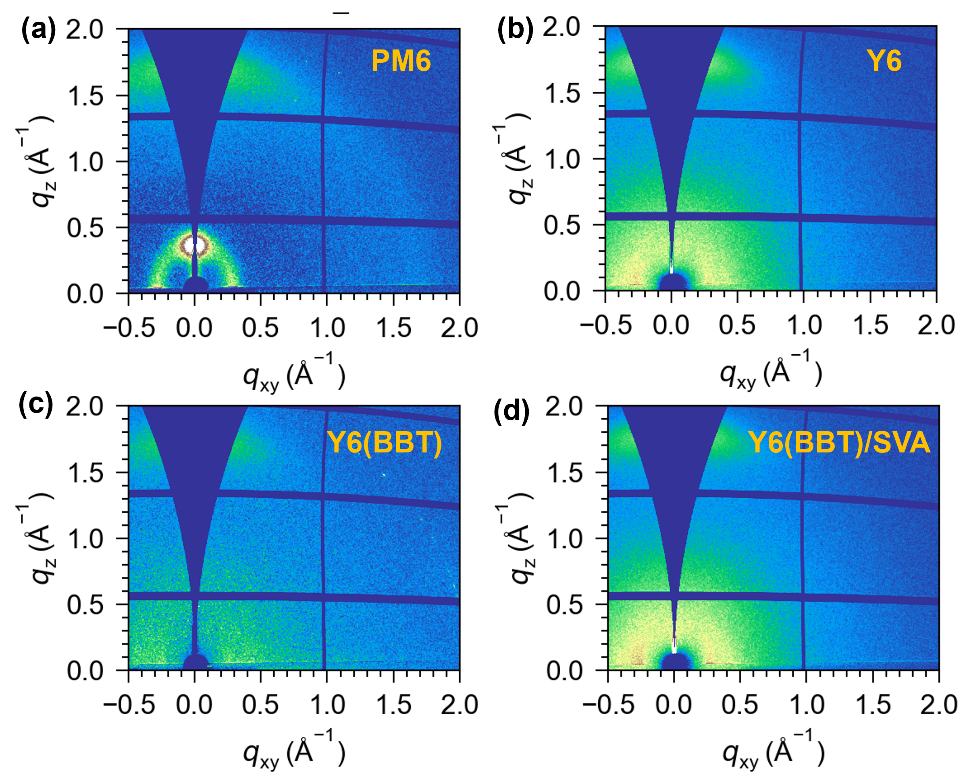


**Figure S10** 2D GIWAXS patterns of (a) PM6, (b) Y6, (c) Y6(BBT), and (d) Y6(BBT)/SVA neat films.


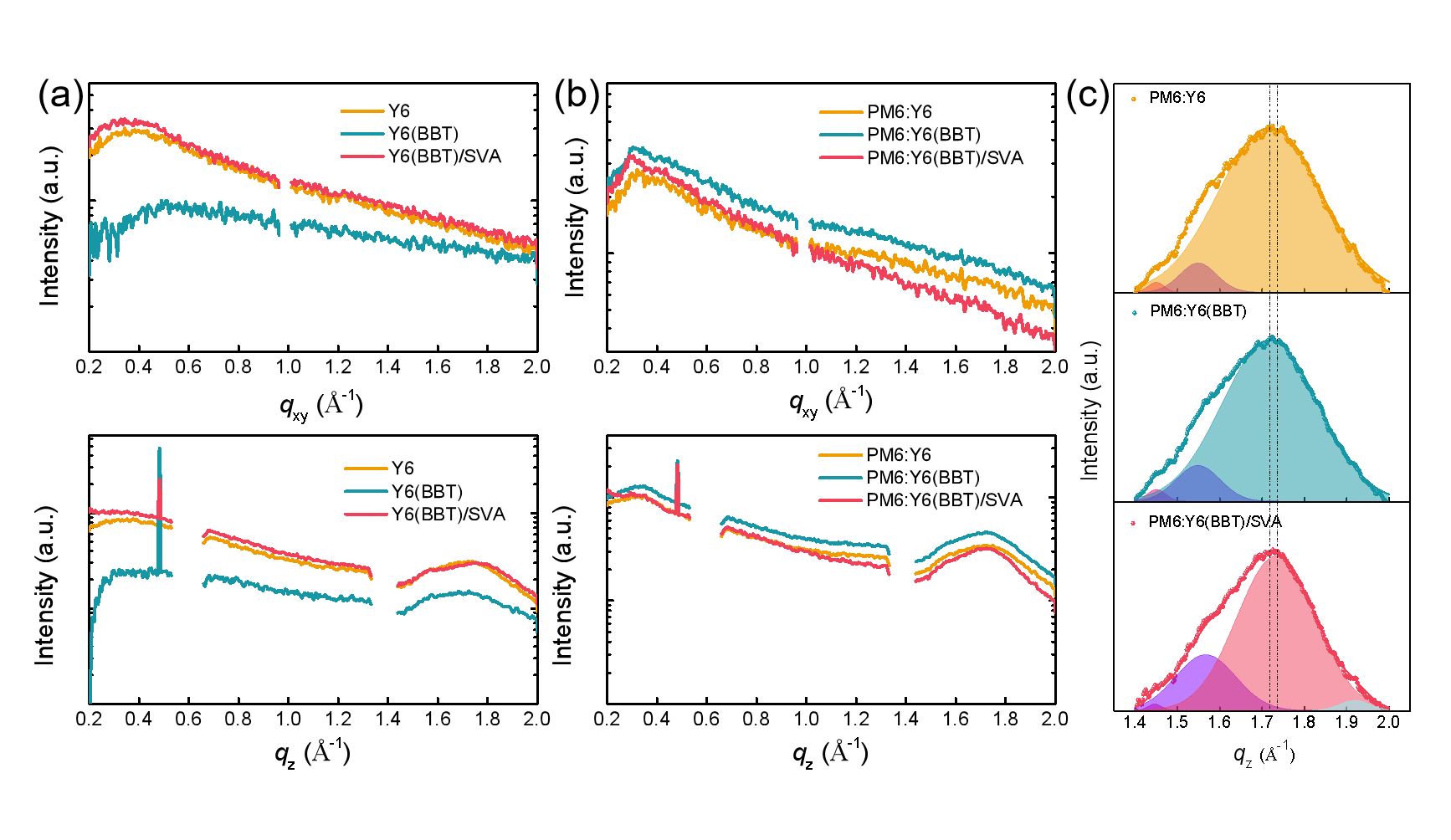


**Figure S11** In-plane (IP) and out-of-plane (OOP) line cuts of (a) Y6, Y6(BBT), and Y6(BBT)/SVA neat films and (b) PM6:Y6, PM6:Y6(BBT), and PM6:Y6(BBT)/SVA blend films. (c) *π*-*π* stacking peak of PM6:Y6, PM6:Y6(BBT), and PM6:Y6(BBT)/SVA blend films in the OOP direction.


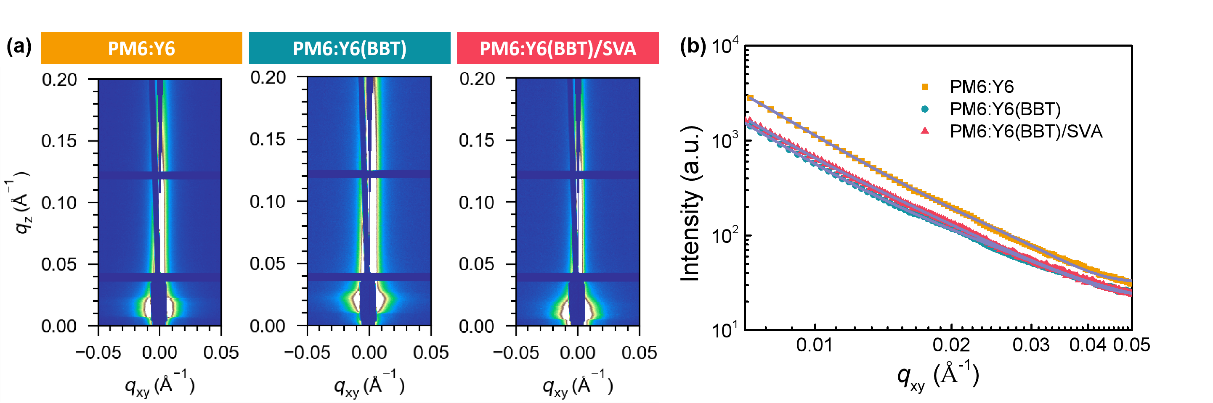


**Figure S12** (a) 2D GISAXS patterns and (b) 1D GISAXS profiles along in-plane direction of PM6:Y6, PM6:Y6(BBT), and PM6:Y6(BBT)/SVA blend films.


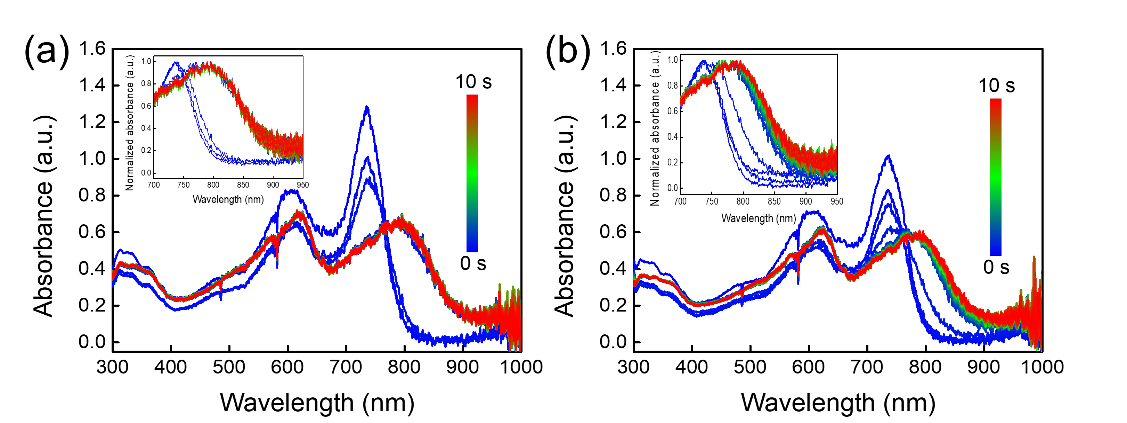


**Figure S13** *in-situ* UV-Vis absorption spectra of (a)PM6:Y6 and (b)PM6:Y6(BBT) blend films. The insets show the zoomed-in UV-Vis spectra.


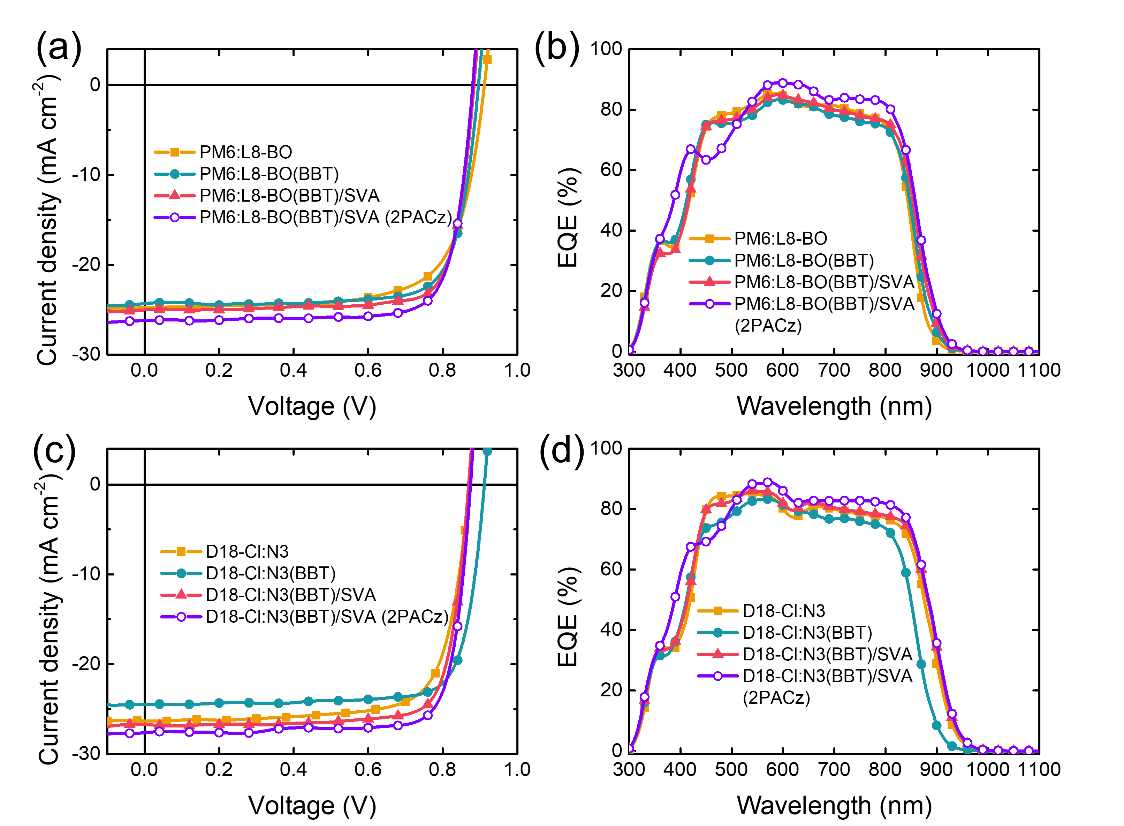


**Figure S14** (a) *J*-*V* curves and (b) corresponding EQE spectra of PM6:L8-BO OSCs. (c) *J*-*V* curves and (d) corresponding EQE spectra of D18-Cl:N3 OSCs.

**Supporting Tables**

**Table S1** Optimization of BBT content for PM6:Y6 conventional solar cells

| BBT  [mg/mL] | *V*_oc_  [V] | *J*_sc_  [mA cm^-2^] | FF  [%] | PCE  [%]*^a^* |
| --- | --- | --- | --- | --- |
| 0 | 0.860 | 25.73 | 70.4 | 15.58 (15.30±0.24) |
| 5 | 0.866 | 25.17 | 75.5 | 16.46 (16.16±0.20) |
| 10 | 0.865 | 25.57 | 77.0 | 17.04 (16.88±0.11) |
| 15 | 0.856 | 24.97 | 76.9 | 16.43 (16.21±0.19) |
| 20 | 0.832 | 24.69 | 77.4 | 15.90 (15.61±0.27) |

*^a^*Average values and standard deviation were obtained from 12 individual devices.

**Table S2** Optimization of SVA time for PM6:Y6(BBT) conventional solar cells

| Time  [min] | *V*_oc_  [V] | *J*_sc_  [mA cm^-2^] | FF  [%] | PCE  [%]*^a^* |
| --- | --- | --- | --- | --- |
| 0 | 0.865 | 25.57 | 77.0 | 17.04 (16.88±0.11) |
| 1 | 0.851 | 26.03 | 77.8 | 17.22 (16.90±0.29) |
| 2 | 0.835 | 26.50 | 79.2 | 17.53 (17.22±0.17) |
| 5 | 0.834 | 26.63 | 78.1 | 17.34 (17.01±0.25) |
| 10 | 0.831 | 26.30 | 77.2 | 16.87 (16.47±0.25) |
| 2*^b^* | 0.836 | 26.84 | 74.3 | 16.68 (16.39±0.24) |
| 2*^c^* | 0.845 | 27.01 | 78.9 | 18.01 (17.66±0.19) |

*^a^*Average values and standard deviation were obtained from 12 individual devices;

*^b^*The active layer was prepared without BBT additives;

*^c^*The device prepared by using 2PACz as anode interface layer (AIL).

**Table S3** Exciton dissociation and charge collection probability of the PM6:Y6-based devices.

| Active layer | *P*_diss_ | *P*_coll_ |
| --- | --- | --- |
| PM6:Y6 | 97.9% | 84.0% |
| PM6:Y6(BBT) | 98.3% | 89.6% |
| PM6:Y6(BBT)/SVA | 98.7% | 91.8% |

**Table S4** Hole and electron mobility.

| Blend film | *μ*_h_  [cm^2^ V^-1^ s^-1^] | *μ*_e_  [cm^2^ V^-1^ s^-1^] | *μ*_h_/*μ*_e_ |
| --- | --- | --- | --- |
| PM6:Y6 | 0.70×10^-4^ | 0.33×10^-4^ | 2.12 |
| PM6:Y6(BBT) | 1.15×10^-4^ | 0.70×10^-4^ | 1.64 |
| PM6:Y6(BBT)/SVA | 1.76×10^-4^ | 1.97×10^-4^ | 0.89 |

**Table S5** Summary detailed GIWAXS data of (010) peak in OOP direction

| Films | *q*  [Å^-1^] | *d*-spacing  [Å] | FWHM  [Å^-1^] | CCL  [Å] |
| --- | --- | --- | --- | --- |
| Y6 | 1.709 | 3.68 | 0.310 | 18.24 |
| Y6(BBT) | 1.706 | 3.68 | 0.291 | 19.43 |
| Y6(BBT)/SVA | 1.763 | 3.56 | 0.243 | 23.27 |
| PM6:Y6 | 1.719 | 3.66 | 0.286 | 19.77 |
| PM6:Y6(BBT) | 1.719 | 3.66 | 0.266 | 21.26 |
| PM6:Y6(BBT)/SVA | 1.735 | 3.62 | 0.219 | 25.82 |

**Table S6** Fitting parameters of 1D GISAXS profile for PM6:Y6 blend films cast at different conditions

| Films | *ξ*  [nm] | *η*  [nm] | D | 2*R*_g_  [nm] |
| --- | --- | --- | --- | --- |
| PM6:Y6 | 25.3 | 6.2 | 3.4 | 33.9 |
| PM6:Y6(BBT) | 30.5 | 5.6 | 3.3 | 29.8 |
| PM6:Y6(BBT)/SVA | 34.1 | 6.8 | 2.7 | 30.4 |

**Table S7** Summary of recently reported high-performance binary OSCs.

| Active layer | *V*_oc_  [V] | *J*_sc_  [mA cm^-2^] | FF  [%] | PCE  [%] | Reference |
| --- | --- | --- | --- | --- | --- |
| D18/L8-BO | 0.918 | 26.86 | 77.25 | 19.05 | [[5](#_ENREF_5)] |
| PBQx-TF:eC9-2Cl | 0.879 | 27.2 | 80.4 | 19.2 | [[6](#_ENREF_6)] |
| PM6:BTP-eC9 | 0.861 | 27.88 | 80.39 | 19.31 | [[7](#_ENREF_7)] |
| PM6:L8-BO | 0.90 | 26.3 | 80.7 | 19.1 | [[8](#_ENREF_8)] |
| PM6:L8-BO | 0.893 | 26.59 | 80.03 | 19.01 | [[9](#_ENREF_9)] |
| D18:L8-BO | 0.91 | 26.48 | 80.65 | 19.65 | [[10](#_ENREF_10)] |
| D18/L8-BO | 0.912 | 26.20 | 80.2 | 19.16 | [[11](#_ENREF_11)] |
| PM6/L8-BO | 0.883 | 26.94 | 81.3 | 19.4 | [[12](#_ENREF_12)] |
| PM6/L8-BO | 0.883 | 26.8 | 81.8 | 19.4 | [[13](#_ENREF_13)] |
| PM6:L8-BO | 0.88 | 26.68 | 80.5 | 19.02 | [[14](#_ENREF_14)] |
| D18-Cl:L8-BO | 0.914 | 26.76 | 78.9 | 19.30 | [[15](#_ENREF_15)] |
| D18/L8-BO-D | 0.922 | 27.04 | 77.5 | 19.3 | [[16](#_ENREF_16)] |
| D18:AQx-2F | 0.937 | 26.1 | 80.4 | 19.7 | [[17](#_ENREF_17)] |
| PM6:L8-BO | 0.887 | 26.85 | 80.1 | 19.1 | [[18](#_ENREF_18)] |
| D18:DT-C8Cl | 0.851 | 28.17 | 80.9 | 19.40 | [[19](#_ENREF_19)] |
| D18-Cl:L8-BO-X | 0.893 | 26.78 | 79.6 | 19.04 | [[20](#_ENREF_20)] |
| D2:L8-BO | 0.901 | 26.39 | 80.1 | 19.03 | [[21](#_ENREF_21)] |
| PM6:L8-BO | 0.898 | 26.17 | 80.87 | 19.01 | [[22](#_ENREF_22)] |
| PM6/L8-BO | 0.891 | 26.80 | 79.7 | 19.03 | [[23](#_ENREF_23)] |
| PM6:eC9 | 0.856 | 29.20 | 78.8 | 19.70 | [[24](#_ENREF_24)] |
| PBDB-TF:eC9 | 0.856 | 27.8 | 80.9 | 19.3 | [[25](#_ENREF_25)] |
| D18:L8-BO | 0.922 | 25.70 | 80.27 | 19.02 | [[26](#_ENREF_26)] |
| D18-Fu:L8-BO | 0.900 | 26.63 | 79.7 | 19.11 | [[27](#_ENREF_27)] |
| PiBT:Y6 | 0.87 | 28.2 | 77.3 | 19.0 | [[28](#_ENREF_28)] |
| D18/L8-BO | 0.908 | 26.39 | 81.59 | 19.55 | [[29](#_ENREF_29)] |
| D18:L8-BO | 0.908 | 26.6 | 80.0 | 19.3 | [[30](#_ENREF_30)] |
| D18-Cl:N3 | 0.875 | 27.63 | 80.8 | 19.53 | Our work |

**References**

[1] Z. Chen, J. Ge, W. Song, X. Tong, H. Liu, X. Yu, J. Li, J. Shi, L. Xie, C. Han, Q. Liu and Z. Ge, *Adv. Mater.*, **2024**, *36*, 2406690.

[2] Y. Wu, J. Guo, W. Wang, Z. Chen, Z. Chen, R. Sun, Q. Wu, T. Wang, X. Hao, H. Zhu and J. Min, *Joule*, **2021**, *5*, 1800-1815.

[3] Q. Liu, Y. Jiang, K. Jin, J. Qin, J. Xu, W. Li, J. Xiong, J. Liu, Z. Xiao, K. Sun, S. Yang, X. Zhang and L. Ding, *Sci. Bull.*, **2020**, *65*, 272-275.

[4] Y. Liang, D. Zhang, Z. Wu, T. Jia, L. Lüer, H. Tang, L. Hong, J. Zhang, K. Zhang, C. J. Brabec, N. Li and F. Huang, *Nat. Energy*, **2022**, *7*, 1180-1190.

[5] Y. Wei, Z. Chen, G. Lu, N. Yu, C. Li, J. Gao, X. Gu, X. Hao, G. Lu, Z. Tang, J. Zhang, Z. Wei, X. Zhang and H. Huang, *Adv. Mater.*, **2022**, *34*, e2204718.

[6] J. Wang, Y. Wang, P. Bi, Z. Chen, J. Qiao, J. Li, W. Wang, Z. Zheng, S. Zhang, X. Hao and J. Hou, *Adv. Mater.*, **2023**, *35*, 2301583.

[7] J. Fu, P. W. K. Fong, H. Liu, C. S. Huang, X. Lu, S. Lu, M. Abdelsamie, T. Kodalle, C. M. Sutter-Fella, Y. Yang and G. Li, *Nat. Commun.*, **2023**, *14*, 1760.

[8] X. Song, H. Xu, X. Jiang, S. Gao, X. Zhou, S. Xu, J. Li, J. Yu, W. Liu, W. Zhu and P. Müller-Buschbaum, *Energy Environ. Sci.*, **2023**, *16*, 3441-3452.

[9] M. Dong, S. Chen, L. Hong, J. Jing, Y. Bai, Y. Liang, C. Zhu, T. Shi, W. Zhong, L. Ying, K. Zhang and F. Huang, *Nano Energy*, **2024**, *119*, 109097.

[10] H. Lu, W. Liu, G. Ran, Z. Liang, H. Li, N. Wei, H. Wu, Z. Ma, Y. Liu, W. Zhang, X. Xu and Z. Bo, *Angew. Chem. Int. Ed.*, **2023**, *135*, e202314420.

[11] M. Xiao, Y. Meng, L. Tang, P. Li, L. Tang, W. Zhang, B. Hu, F. Yi, T. Jia, J. Cao, C. Xu, G. Lu, X. Hao, W. Ma and Q. Fan, *Adv. Funct. Mater.*, **2024**, *34*, 2311216.

[12] Z. Gan, L. Wang, J. Cai, C. Guo, C. Chen, D. Li, Y. Fu, B. Zhou, Y. Sun, C. Liu, J. Zhou, D. Liu, W. Li and T. Wang, *Nat. Commun.*, **2023**, *14*, 6297.

[13] L. Wang, C. Chen, Y. Fu, C. Guo, D. Li, J. Cheng, W. Sun, Z. Gan, Y. Sun, B. Zhou, C. Liu, D. Liu, W. Li and T. Wang, *Nat. Energy*, **2024**, *9*, 208-218.

[14] G. Ding, T. Chen, M. Wang, X. Xia, C. He, X. Zheng, Y. Li, D. Zhou, X. Lu, L. Zuo, Z. Xu and H. Chen, *Nano-Micro Lett.*, **2023**, *15*, 92.

[15] Y. Kan, Y. Sun, Y. Ren, Y. Xu, X. Jiang, H. Shen, L. Geng, J. Li, P. Cai, H. Xu, K. Gao and Y. Li, *Adv. Mater.*, **2024**, *36*, 2312635.

[16] Y. Zhu, D. He, C. Wang, X. Han, Z. Liu, K. Wang, J. Zhang, X. Shen, J. Li, Y. Lin, C. Wang, Y. He and F. Zhao, *Angew. Chem. Int. Ed.*, **2024**, *136*, e202316227.

[17] K. Liu, Y. Jiang, G. Ran, F. Liu, W. Zhang and X. Zhu, *Joule*, **2024**, *8*, 835-851.

[18] F. Sun, X. Zheng, T. Hu, J. Wu, M. Wan, Y. Xiao, T. Cong, Y. Li, B. Xiao, J. Shan, E. Wang, X. Wang and R. Yang, *Energy Environ. Sci.*, **2024**, *17*, 1916-1930.

[19] S. Chen, S. Zhu, L. Hong, W. Deng, Y. Zhang, Y. Fu, Z. Zhong, M. Dong, C. Liu, X. Lu, K. Zhang and F. Huang, *Angew. Chem. Int. Ed.*, **2024**, *136*, e202318756.

[20] S. Luo, C. Li, J. Zhang, X. Zou, H. Zhao, K. Ding, H. Huang, J. Song, J. Yi, H. Yu, K. S. Wong, G. Zhang, H. Ade, W. Ma, H. Hu, Y. Sun and H. Yan, *Nat. Commun.*, **2023**, *14*, 6964.

[21] X. Deng, B. Huang, Y. Fang, D. Chen, Y. Cheng, S. Chen, J. Zhang, L. Zhang, S. Jeong, F. Wu, J. Liu, L. Chen, C. Yang and Y. Chen, *Adv. Funct. Mater.*, **2024**, *34*, 2315476.

[22] Z. Wang, H. Wang, M. Du, X. Lai, F. He, Q. Guo, Q. Guo, A. Tang, X. Sun and E. Zhou, *Adv. Funct. Mater.*, **2023**, *34*, 2313240.

[23] W. Wu, Y. Luo, T. A. Dela Peña, J. Yao, M. Qammar, M. Li, H. Yan, J. Wu, R. Ma and G. Li, *Adv. Energy Mater.*, **2024**, *14*, 2400354.

[24] X. Yu, P. Ding, D. Yang, P. Yan, H. Wang, S. Yang, J. Wu, Z. Wang, H. Sun, Z. Chen, L. Xie and Z. Ge, *Angew. Chem. Int. Ed.*, **2024**, *63*, e202401518.

[25] Z. Chen, S. Zhang, T. Zhang, J. Dai, Y. Yu, H. Li, X. Hao and J. Hou, *Joule*, **2024**, *8*, 1723-1734.

[26] Y. Li, Z. Jia, P. Huang, T. Liu, D. Hu, Y. Li, H. Liu, X. Lu, S. Lu, X. Yin and Y. Yang, *Adv. Energy Mater.*, **2024**, *14*, 2304000.

[27] L. Chen, J. Yi, R. Ma, T. A. Dela Peña, Y. Luo, Y. Wang, Y. Wu, Z. Zhang, H. Hu, M. Li, J. Wu, G. Zhang, H. Yan and G. Li, *Mat. Sci. Eng. R*, **2024**, *159*, 100794.

[28] T. Lin, Y. Hai, Y. Luo, L. Feng, T. Jia, J. Wu, R. Ma, T. A. Dela Pena, Y. Li, Z. Xing, M. Li, M. Wang, B. Xiao, K. S. Wong, S. Liu and G. Li, *Adv. Mater.*, **2024**, *36*, 2312311.

[29] M. Xie, L. Zhu, J. Zhang, T. Wang, Y. Li, W. Zhang, Z. Fu, G. Zhao, X. Hao, Y. Lin, H. Zhou, Z. Wei and K. Lu, *Adv. Energy Mater.*, **2024**, *14*, 2400214.

[30] Y. Sun, L. Wang, C. Guo, J. Xiao, C. Liu, C. Chen, W. Xia, Z. Gan, J. Cheng, J. Zhou, Z. Chen, J. Zhou, D. Liu, T. Wang and W. Li, *J. Am. Chem. Soc.*, **2024**, *146*, 12011-12019.
